# A novel biocatalyst for efficient production of 2-oxo-carboxylates using glycerol as the cost-effective carbon source

**DOI:** 10.1186/s13068-015-0368-y

**Published:** 2015-11-25

**Authors:** Yujiao Wang, Yingxin Zhang, Tianyi Jiang, Jingjing Meng, Binbin Sheng, Chunyu Yang, Chao Gao, Ping Xu, Cuiqing Ma

**Affiliations:** State Key Laboratory of Microbial Technology, Shandong University, Jinan, 250100 People’s Republic of China; State Key Laboratory of Microbial Metabolism, School of Life Sciences and Biotechnology, Shanghai Jiao Tong University, Shanghai, 200240 People’s Republic of China

**Keywords:** Glycerol, *Pseudomonas putida* KT2440, Biocatalyst, NAD-independent lactate dehydrogenase, 2-Oxo-carboxylate

## Abstract

**Background:**

The surplus of glycerol has increased remarkably as a main byproduct during the biofuel’s production. Exploiting an alternative route for glycerol utilization is significantly important for sustainability of biofuels.

**Results:**

A novel biocatalyst that could be prepared from glycerol for producing 2-oxo-carboxylates was developed. First, *Pseudomonas putida* KT2440 was reconstructed by deleting *lldR* to develop a mutant expressing the NAD-independent lactate dehydrogenases (iLDHs) constitutively. Then, the *Vitreoscilla* hemoglobin (VHb) was heterologously expressed to further improve the biotransformation activity. The reconstructed strain, *P. putida* KT2440 (Δ*lldR*)/pBSPPc_Gm_-*vgb*, exhibited high activities of iLDHs when cultured with glycerol as the carbon source. This cost-effective biocatalyst could efficiently produce pyruvate and 2-oxobutyrate from dl-lactate and dl-2-hydroxybutyrate with high molar conversion rates of 91.9 and 99.8 %, respectively.

**Conclusions:**

The process would not only be a promising alternative for the production of 2-oxo-carboxylates, but also be an example for preparation of efficient biocatalysts for the value-added utilization of glycerol.

## Background

The increasing demand for renewable fuel has resulted in the increasing production of biofuels. As an inevitable byproduct, glycerol is generated in both bioethanol and biodiesel production processes [1]. For instance, a large amount of glycerol is produced during fermentative production of bioethanol [1]. About 10 kg of crude glycerol will be generated from every 100 kg of biodiesel production by the transesterification of fats and oils with alcohol [2]. Significant amounts of glycerol surplus, created by the increasing bioethanol and biodiesels production, have given rise to a sharp drop in glycerol price [1–3]. The painfully low glycerol price has negatively impacted the development of biofuels industry [2]. Therefore, exploiting new value-added alternate ways of glycerol utilization is positive to improve the viability of the biofuel economy.

Glycerol can be converted into various high-value products via either chemical transformations or biological conversions [4, 5]. For example, high-value fine chemicals such as dihydroxyacetone, tartronic acid, and mesoxalic acid can be obtained by selective catalytic oxidation of glycerol or glycerol derivatives [6]. Due to its advantages of higher specificity, milder reaction conditions, and lower levels of chemical contaminants, biological conversion is more desirable for environment-friendly production of those valuable chemicals from glycerol [3, 4, 7].

A wide variety of microorganisms, including the *Pseudomonas putida* KT2440, are able to utilize glycerol as the carbon and energy source [8, 9]. Recent studies related to biotechnological production of fuels and chemicals from glycerol have focused on metabolites derived from glycerol-assimilation process such as 1,3-propanediol, succinic acid, 2,3-butanediol, and ethanol [10–13]. There are also some studies concerning the use of glycerol for cultures of the oil-accumulating microbes such as microalgae, bacteria, yeasts and other fungi. The lipids recovered from whole cells of these oil-accumulating microbes can be transesterified into biodiesel and possess a composition similar to that of plant-based oils [14–16].

Besides the source of lipids, whole cells of microbes with different enzymes could also be used in the biocatalysis processes [17]. For example, there is a large amount of potential enzymes in genome of *P. putida* KT2440, which evolutionarily endow this strain with capacities to host a variety of biodegradation pathways and enable it to withstand exposure to diverse types of aggressive aromatic compounds, such as naphthalene, 4-chloronitrobenzene, 2,4-xylenol and phenol [18]. *P. putida* KT2440 is a microorganism officially classified as Generally Recognized as Safe. Thus, the whole cells of the strain can be used as a robust biocatalyst for biotransformation in various applications. However, these potential enzymes, such as NAD-independent lactate dehydrogenases (iLDHs, which involved in the catabolism of lactate) [19] and the enzymes involved in the catabolism of catechol (encoded by a *cat* cluster) [20] are generally strictly regulated, requiring expensive or toxic inducers for their expressions.

Since the release of complete sequence of *P. putida* KT2440’s genome, metabolic networks of the *P. putida* KT2440 have been reconstructed and the regulation mechanisms of various potential enzymes have been disclosed. Here, we reported a case study of production of 2-oxo-carboxylates, using glycerol as the carbon source for the biocatalyst preparation, which based on the regulation mechanism of lactate utilization [19]. The transcriptional repressor LldR of lactate utilization operon was deleted in the *P. putida* KT2440. Whole cells of the recombinant strain were confirmed to constitutively exhibit the activities of iLDHs catalyzing the oxidation of 2-hydroxy-carboxylates. Then, two important 2-oxo-carboxylates: pyruvate and 2-oxobutyrate (2-OBA) were produced with whole cells of the recombinant strain prepared from glycerol as the catalyst. The process provides a promising alternative for the value-added utilization of biotechnologically produced glycerol.

## Results and discussion

### Regulatory networks of glycerol and lactate metabolism in *P. putida* KT2440

**Fig. 1:**
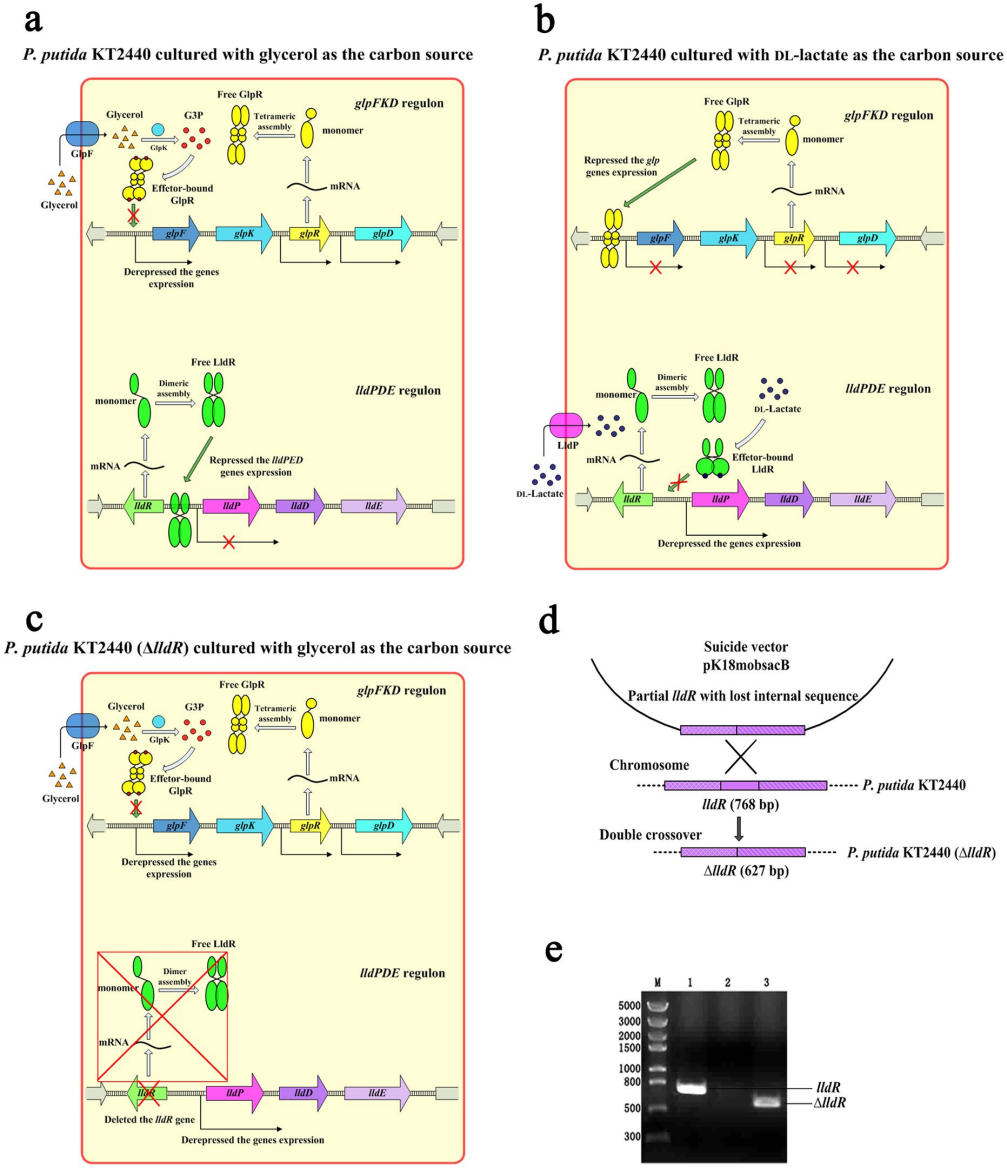
The hypothetic schematics of regulatory networks of glycerol and lactate metabolism in *P. putida* KT2440, and the construction of *P. putida* KT2440 (Δ*lldR*). **a** The derepression of the *glp* genes expression is occurring when the strain cultured in MSM with glycerol as carbon source. On the other hand, the expressions of *lldPDE* genes are repressed by the LldR, without the lactate as inducer. **b** The derepression of the *lldPDE* genes expression is occurring when the strain cultured in MSM with dl-lactate as carbon source. On the contrary, the free tetrameric assembly of GlpR will tightly bind to the DNA operator sequence with the DNA-binding domains, and inhibit the expressions of *glp* genes. **c** If the *lldR* gene is deleted, the repression of the *lldPDE* genes expression will be damaged, owing to the absence of the functional LldR. And the *lldPDE* genes will still fully express, even without lactate as the inducer. **d** Diagram illustrating the disruption of the *lldR* mediated by homologous double crossover. **e** Analysis of PCR fragments to confirm *lldR* disruption. *Lane M* molecular mass standard (λDNA/HindIII); *lane 1* product amplified with *P. putida* KT2440 genomic DNA as the template; *lane 2* product amplified with water as the template (negative control); *lane 3* product amplified with *P. putida* KT2440 (Δ*lldR*) genomic DNA as the template. The PCRs were performed with primers *lldR*k.f and *lldR*k.r

Owing to its versatile metabolic activities, *P. putida* KT2440 can use various organics as carbon and energy sources, which make this strain an ideal industrial microorganism used for biotransformation and biodegradation [21]. However, most of the metabolism networks are subject to the strict regulation. Take glycerol and lactate as examples, both of these two compounds can be used as carbon and energy sources for *P. putida* KT2440 [9, 22]. During the glycerol utilization process, the specific enzymes related to the glycerol metabolism, including GlpF (a glycerol transporter encoded by *glpF*), GlpK (a glycerol kinase encoded by *glpK*), and GlpD (a glycerol-3-phosphate dehydrogenase encoded by *glpD*), will be generally induced [9]. The expressions of these genes are regulated by GlpR, a DeoR family transcriptional regulator, which is encoded by *glpR* (PP1074) [9, 23, 24]. The expression of *glpR* is not affected by glycerol [9]. The primary structure and N-terminal helix-turn-helix (HTH) DNA-binding motif of GlpR in *Escherichia coli* K12 have already been identified [25]. Moreover, previous research has shown that the GlpR of *E. coli* K12 is a tetramer under native conditions [26]. Since the GlpR from *E. coli* K12 and *P. putida* KT2440 share the high consensus positions (71.0 %) and identity positions (54.8 %), we conjectured that the GlpR from *P. putida* KT2440 might be a tetramer and possess N-terminal HTH DNA-binding motifs as well. The structure of C-terminal effector-binding domain of DeoR from *Bacillus subtilis* has been determined [27]. In view of the fact that the GlpR belongs to the DeoR family transcriptional regulator [25], the GlpR might also possess the similar C-terminal effector-binding domains. Based on these backgrounds mentioned above, the hypothetic schematics of the regulatory networks of the GlpR-depended glycerol metabolism in *P. putida* KT2440 are shown in Fig. 1. As shown in Fig. 1a, when *P. putida* KT2440 is cultured with the glycerol as a carbon source, the glycerol from the extracellular environment will be transported into cytoplasm (mediated by GlpF) [24]. The sn-glycerol-3-P (G3P) produced by the substrate phosphorylation of glycerol (mediated by GlpK, the glycerol kinase) will be generated via the effector-independent expression of *glp* genes, which occurs in a low-probability stochastic way [24]. And then the repression of *glp* genes mediated by GlpR will be derepressed by the increasing intracellular G3P [24]. As an effector for GlpR, G3P can bind to the effector-binding domains of GlpR, and then will prevent the HTH DNA-binding domains of GlpR from binding to the DNA operator sequence of *glp* regulon [24, 25, 27]. On the contrary, the free tetrameric assembly of GlpR will tightly bind to the DNA operator sequence and inhibit the expressions of *glp* genes when the strain cultured without glycerol (Fig. 1b) [24, 27].

The similar regulation network also exists in the lactate metabolism of *P. putida* KT2440. The *lldPDE* operon, which is responsible for lactate utilization in most *Pseudomonas* strains including *P. putida* KT2440, has been studied in previous reports [19, 28–31]. The *lldPDE* operon comprises 3 genes, *lldP* (encoding a lactate permease), *lldD* (encoding an l-iLDH), and *lldE* (encoding a d-iLDH) [19, 22]. The l-iLDH and d-iLDH are mainly responsible for the oxidation of l-lactate and d-lactate to pyruvate, respectively [19, 28, 31]. However, the expressions of *lldD* and *lldE* are both repressed by the regulator LldR which is encoded by upstream adjacent gene *lldR* [19]. The previous study reported that the LldR from *Corynebacterium glutamicum* is a homodimer assembled by domain swapping [32]. The N-domain of this regulator contains a typical winged HTH (WHTH) DNA-binding domain [32, 33]. And the C-terminal domain is assumed to play the ligand-binding role [32]. As the primary structure predicted by NCBI, the monomer of LldR from *P. putida* KT2440 comprises an N-terminal WHTH DNA-binding and a C-terminal ligand-binding domain, which is coincident with the typical region features of many members of the GntR family [34]. The hypothetic schematics of the regulatory networks of the LldR-mediated lactate metabolism in *P. putida* KT2440 have also been shown in Fig. 1. As shown in Fig. 1a, while the strain cultured in the medium without lactate, the free LldR homodimer can bind to the promoter region of the *lldPDE* operon with the WHTH DNA-binding domains, and will inhibit the expressions of *lldPDE* genes downstream. However, the derepression of LldR to the *lldPDE* genes is occurring when lactate exists in the growing environment of the strain. As an effector of LldR, the lactate can bind to the C-terminal ligand-binding domains. Then, the effector-bound LldR will lose the ability of binding to the DNA promoter sequence of *lldPDE* regulon (Fig. 1b).

### Reconstruction of *P. putida* KT2440 *lldR* deletion mutant

As mentioned before, the presence of lactate is necessary for the expressions of l-iLDH and d-iLDH [19, 31, 35]. As a result, the hyperosmotic medium caused by high concentration of lactate becomes a major limitation for high-density culture, and the indispensable lactate addition raises the cost of biocatalysts preparation. Considering the versatile applications of *P. putida* KT2440 in biocatalysis, it is rather desirable to prepare efficient biocatalyst from a more cost-effective substrate, such as glycerol, with the required enzymes iLDHs. To achieve this goal, the regulatory network of lactate utilization was reconstructed. After deleting the *lldR* gene, it seems likely that the regulatory network would be broken down because of the absence of the functional LldR. Therefore, when cells incubated with the glycerol as the sole carbon source, the *lldPDE* genes would still fully express, even without the induction of lactate (Fig. 1c). To explore this possibility, we disrupted the *lldR* gene which encodes negative regulator LldR in *P. putida* KT2440. The suicide plasmid pK18*mobsacB* which mediated the homologous recombination was used for deleting the *lldR* gene (Fig. 1d) [36]. The disruption of the gene *lldR* was verified by PCR (Fig. 1e). The result strain is named *P. putida* KT2440 (Δ*lldR*).

### Effect of inactivation of *lldR* on iLDHs expression

The wild-type *P. putida* KT2440 and *P. putida* KT2440 (Δ*lldR*) were cultured in 500-mL baffled shake flasks each containing 100 mL minimal salt medium (MSM) [31] supplied with 5 g/L dl-lactate or glycerol as the carbon source. And 1 mM octanoate, as the co-feeder, was added to the MSM with glycerol to shorten lag phase [23]. To investigate the effect of inactivation of *lldR* on iLDHs expression, the activities of l-iLDH and d-iLDH in crude cell extracts of *P. putida* KT2440 and *P. putida* KT2440 (Δ*lldR*) were assayed, with 2,6-dichloroindophenol (DCIP) as the artificial electron acceptor and 20 mM l- or d-lactate as the electron donor. As shown in Table 1, when the *P. putida* KT2440 was cultured in the medium with dl-lactate as the sole carbon source, the enzymes activities of l-iLDH and d-iLDH were 161.4 nmol/min mg protein and 332.9 nmol/min mg protein, respectively. However, neither l-iLDH nor d-iLDH activity was detectable in *P. putida* KT2440 when cultured with glycerol. Comparatively, when the *P. putida* KT2440 (Δ*lldR*) was incubated in the MSM with dl-lactate as the carbon source, the activity of l-iLDH was 220.9 nmol/min mg protein and d-iLDH was 471.5 nmol/min mg protein. While incubated in the MSM containing the glycerol as the carbon source, this Δ*lldR* mutant also exhibited high activities of iLDHs, 348.2 nmol/min mg protein and 771.0 nmol/min mg protein for l-iLDH and d-iLDH, respectively. These results revealed that the repression effects of LldR on l-iLDH and d-iLDH expressions were removed by the disruption of *lldR* gene. When cultured with glycerol, the iLDHs activities of the *P. putida* KT2440 (Δ*lldR*) were not impaired, compared with that cultured with dl-lactate. Therefore, the *P. putida* KT2440 (Δ*lldR*), in which l-iLDH and d-iLDH are expressed constitutively, has the potential to efficiently produce pyruvate from lactate with glycerol as the cost-effective culture substrate.

**Table 1:** Activities of iLDHs in crude cell extracts of *P. putida* KT2440 and *P. putida*. KT2440 (∆*lldR*) cultured with different growth substrates

Growth substrate	Strain	Enzyme activity (nmol/min mg protein)^a^	
		l-iLDH	d-iLDH
dl-Lactate	*P. putida* KT2440	161.4 ± 4.6	332.9 ± 4.3
	*P. putida* KT2440 (∆*lldR*)	220.9 ± 7.0	471.5 ± 3.5
Glycerol	*P. putida* KT2440	ND	ND
	*P. putida* KT2440 (∆*lldR*)	348.2 ± 11.2	771.0 ± 9.0

*ND* not detected

^a^Activities of d-iLDH and l-iLDH were examined with 20 mM d-lactate or 20 mM l-lactate. DCIP was used as the electron acceptor. Results are mean ± SD of three parallel replicates

### *Vitreoscilla* hemoglobin (VHb) enhances the lactate oxidation

It has been revealed in a previous study that iLDHs from *P. stutzeri* SDM could not oxidize lactate with oxygen as the directly electron acceptor [37]. The *lldPDE* operon organization is similar in *P. putida* KT2440 and *P. stutzeri* SDM, and the lactate utilization genes between these two strains show strikingly high homology [19, 29, 38]. It is inferred that the electron produced in the lactate oxidation process might terminally transfer to the oxygen, a final electron acceptor, through the electron transport chain in *P. putida* KT2440, as well as in *P. stutzeri* SDM [37].

**Fig. 2:**
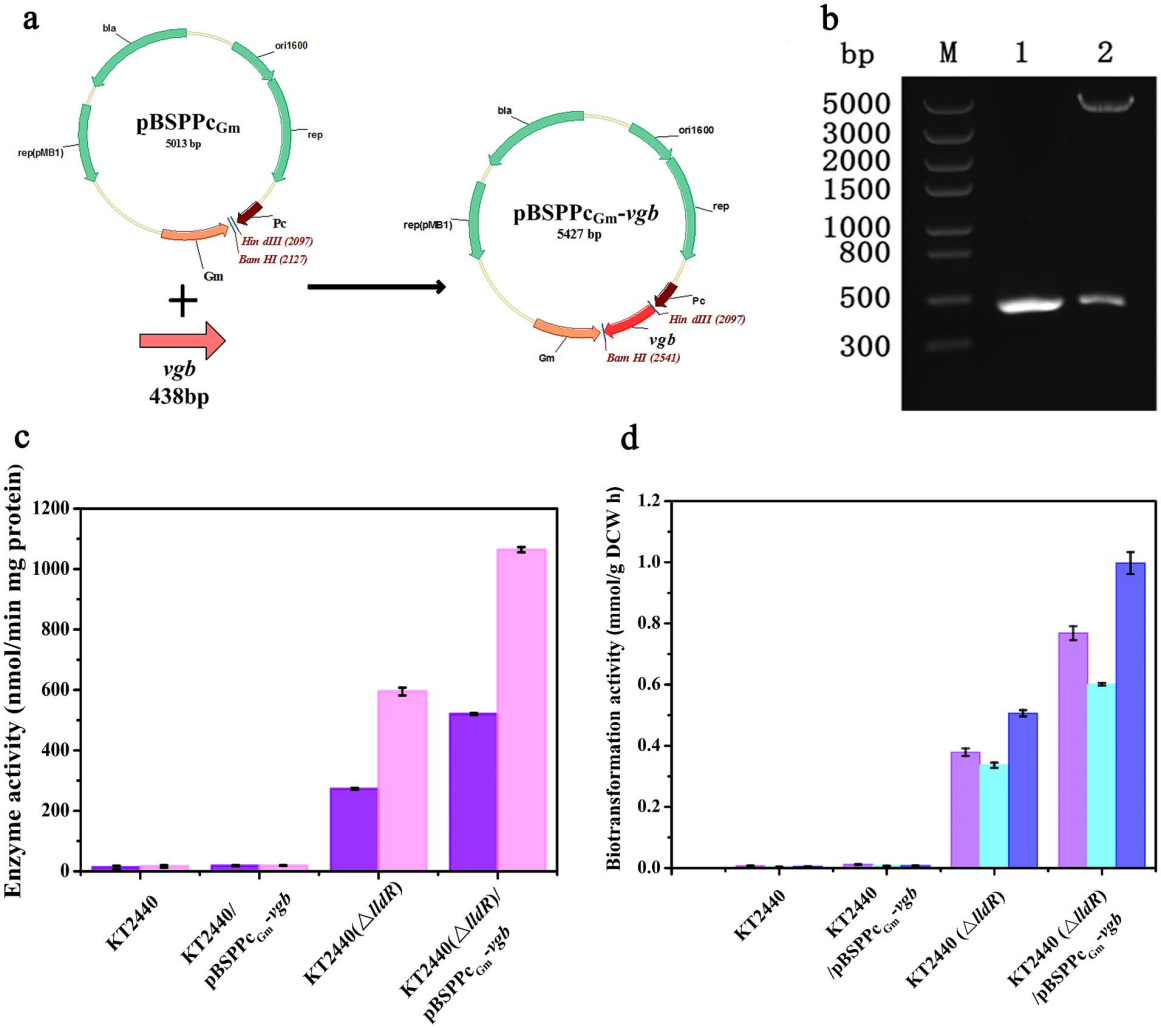
Construction of the *vgb* expressing vector and comparisons of iLDHs activities and biotransformation rates toward pyruvate production between different *P. putida* KT2440 strains. **a** Construction of recombinant vector pBSPPc_Gm_-*vgb*. *vgb*, the gene encoding VHb. pBSPPc_Gm_, a broad-host-range constitutive vector containing a *P*_c_ promoter. The *vgb* gene was inserted into the pBSPPc_Gm_-*vgb* in the corresponding sites, to generate the plasmid pBSPPc_Gm_-*vgb*. **b** Verification of pBSPPc_Gm_-*vgb. Lane M* molecular mass standard (λDNA/HindIII); *lane 1* product amplified with pET28b-RgDAAO-VHb as the template; *lane 2* double enzymes digestion (*HindIII* and *BamHI*) of recombinant vector pBSPPc_Gm_-*vgb*. **c** Activities of l-iLDH (*violet bars*) and d-iLDH (*light magenta bars*) in crude cell extracts of *P. putida* KT2440 and its derivatives were examined with DCIP as the artificial electron acceptor and 20 mM l- or d-lactate as the electron donor. Results are mean ± SD of three parallel replicates. **d** The biotransformation rates of pyruvate production by whole cells of different *P. putida* KT2440 strains. The biotransformations were conducted with the whole cells of *P. putida* KT2440, *P. putida* KT2440/pBSPPc_Gm_-*vgb*, *P. putida* KT2440 (Δ*lldR*) and *P. putida* KT2440 (Δ*lldR*)/pBSPPc_Gm_-*vgb* with 100 mM l-lactate (*violet bars*), 100 mM d-lactate (*light cyan bars*) and 100 mM dl-lactate (*blue bars*) as the substrates. The concentrations of the pyruvate were measured by HPLC. Results are mean ± SD of three parallel replicates

*Vitreoscilla* hemoglobin (VHb) is a soluble homodimeric globin encoded by *vgb*, a 438 bp gene discovered in *Vitreoscilla* sp. [39]. It is the first bacterial hemoglobin whose structure and function have been well characterized [39, 40]. Since the gene (*vgb*) encoding VHb has been cloned [41, 42], its heterologous expression has become an engineering strategy widely used to increase production of a diverse of bioproducts and facilitate the bioremediation [43]. In this study, the *vgb* gene was amplified with the primers *vgb.f* and *vgb.r* from the vector pET28b-RgDAAO-VHb. Then, the 438 bp *vgb* fragment was ligated to *Hind*III and *BamH*I double-digested pBSPPc_Gm_, a broad-host-range constitutive vector containing a *P*_c_ promoter [44], to produce pBSPPc_Gm_-*vgb* (Fig. 2a). As shown in Fig. 2b, the *vgb* gene has been successfully cloned and inserted into the pBSPPc_Gm_ with the corresponding restriction enzyme sites, obtaining the pBSPPc_Gm_-*vgb*. The vector was transferred into *P. putida* KT2440 and *P. putida* KT2440 (Δ*lldR*) by electroporation to produce *P. putida* KT2440/pBSPPc_Gm_-*vgb* and *P. putida* KT2440 (Δ*lldR*)/pBSPPc_Gm_-*vgb*, respectively.

To explore the effect of introduction of VHb in lactate oxidation, the l-iLDH and d-iLDH activities in the crude cell extracts of *P. putida* KT2440, *P. putida* KT2440 (Δ*lldR*), *P. putida* KT2440/pBSPPc_Gm_-*vgb* and *P. putida* KT2440 (Δ*lldR*)/pBSPPc_Gm_-*vgb*, cultured with glycerol, were assayed. As the results shown in Fig. 2c, the introduction of VHb did not increase the activities of iLDHs in *P. putida* KT2440 cultured with the glycerol as carbon source. The enzymes activities of l-iLDH and d-iLDH in *P. putida* KT2440 (Δ*lldR*) were 273.0 nmol/min mg protein and 595.0 nmol/min mg protein, respectively. However, compared with *P. putida* KT2440 (Δ*lldR*), both the activities of l-iLDH and d-iLDH in *P. putida* KT2440 (Δ*lldR*)/pBSPPc_Gm_-*vgb* significantly increased to 520.7 nmol/min mg protein and 1063.9 nmol/min mg protein, respectively, almost twofold higher than those in *P. putida* KT2440 (Δ*lldR*).

### Effect of VHb introduction in whole-cell biocatalysis

It was also investigated if the introduction of VHb could affect the whole cells biocatalysis activities. The biocatalysis reactions were conducted at 30 °C in phosphate buffer (pH 7.4) for 6 h, with 10.5 g dry cell weight (DCW)/L of *P. putida* KT2440, *P. putida* KT2440/pBSPPc_Gm_-*vgb*, *P. putida* KT2440 (Δ*lldR*) and *P. putida* KT2440 (Δ*lldR*)/pBSPPc_Gm_-*vgb*, which cultured with glycerol, as the biocatalysts, respectively. l-Lactate, d-lactate, and racemic lactate at 100 mM were used as the substrates. The biocatalysis reactions were carried out in the presence of 30 mM ethylenediaminetetraacetic acid (EDTA), which could remove bivalent ions necessary for 2-keto-acid decarboxylase-catalyzed reactions [45, 46], and then could block the degradation of 2-oxo-carboxylates. As shown in Fig. 2d, via 6 h biotransformation, no pyruvate production was detected when either *P. putida* KT2440 or *P. putida* KT2440/pBSPPc_Gm_-*vgb* was used, which was in correspondence to the result of the activities assays of iLDHs of these two strains. However, both *P. putida* KT2440 (Δ*lldR*) and *P. putida* KT2440 (Δ*lldR*)/pBSPPc_Gm_-*vgb* have the ability to oxidize the two enantiomers of lactate. For *P. putida* KT2440 (Δ*lldR*), the oxidation rates toward l-lactate, d-lactate and dl-lactate to pyruvate were 0.38 mmol/g DCW h, 0.34 mmol/g DCW h and 0.51 mmol/g DCW h, respectively. And for *P. putida* KT2440 (Δ*lldR*)/pBSPPc_Gm_-*vgb*, the oxidation rates toward these three kinds of lactate have remarkably increased to 0.77 mmol/g DCW h, 0.60 mmol/g DCW h and 1.00 mmol/g DCW h, respectively (Fig. 2d). As expected, the reconstructed strain with heterologously expressed VHb exhibited about twofold higher biotransformation activities than the strain without VHb expression. These results revealed that the introduction of VHb into the recombinant *P. putida* KT2440 (Δ*lldR*) indeed significantly enhanced the whole cells biocatalysis activities of lactate oxidation to produce pyruvate. Furthermore, the pyruvate production rates from dl-lactate were significantly higher than which from either l-lactate or d-lactate alone (Fig. 2d). l-iLDH and d-iLDH catalyze the oxidation of l-lactate and d-lactate, respectively. The higher biotransformation activity toward dl-lactate might be due to the fact that both isomers in dl-lactate would be simultaneously oxidized by l-iLDH and d-iLDH in these recombinant strains. Compared with optical pure lactate, the low price and large sources of racemic lactate make it become a more cost-effective substrate to produce pyruvate. Based on the results above, the *P. putida* KT2440 (Δ*lldR*)/pBSPPc_Gm_-*vgb*, a recombinant strain with constitutive iLDHs and heterologously expressed VHb, has the potential to efficiently produce 2-oxo-carboxylates from 2-hydroxy-carboxylates.

### Pyruvate and 2-OBA production through whole-cell biocatalysis

The oxidation of two most important members of 2-hydroxy-carboxylates, lactate and 2-hydroxybutyrate (2-HBA) that are catalyzed by iLDHs, have been studied in previous studies [37, 47]. In this study, the biocatalytic oxidation of racemic lactate (100 mM) and 2-HBA (100 mM) were carried out, with 10.5 g DCW/L of whole cells of *P. putida* KT2440, *P. putida* KT2440/pBSPPc_Gm_-*vgb*, *P. putida* KT2440 (Δ*lldR*) and *P. putida* KT2440 (Δ*lldR*)/pBSPPc_Gm_-*vgb*, which were prepared from glycerol, as the biocatalysts, respectively. The biocatalysis reactions were conducted at 30 °C in phosphate buffer (pH 7.4) with the presence of 30 mM EDTA. As shown in Tables 2 and 3, the racemic lactate (100 mM) and 2-HBA (100 mM) were completely oxidized into pyruvate and 2-OBA, via 6-h bioconversion with *P. putida* KT2440 (Δ*lldR*)/pBSPPc_Gm_-*vgb* as the biocatalyst. The yields of pyruvate and 2-OBA with *P. putida* KT2440 (Δ*lldR*) were 50.9 and 74.7 %, respectively. However, the yields of these two productions with *P. putida* KT2440 (Δ*lldR*)/pBSPPc_Gm_-*vgb* were 91.9 and 99.8 %, respectively, about 1.8-fold and 1.3-fold higher than which catalyzed by *P. putida* KT2440 (Δ*lldR*). The final concentrations of pyruvate and 2-OBA produced by *P. putida* KT2440 (Δ*lldR*)/pBSPPc_Gm_-*vgb* were 90.9 mM and 99.3 mM, respectively.

**Table 2:** Comparison of pyruvate productions by whole cells of *P. putida* KT2440, *P. putida*/pBSPPc_Gm_-*vgb*, *P. putida* KT2440 (Δ*lldR*), and *P. putida* KT2440 (Δ*lldR*)/pBSPPc_Gm_-*vgb*

Strain	Pyruvate (mM)	Yield (%)^a^	Productivity (mmol/g DCW h)
KT2440	0	0	0
KT2440/pBSPPc_Gm_-*vgb*	0	0	0
KT2440 (Δ*lldR*)	50.60 ± 0.38	50.9	0.81 ± 0.005
KT2440 (Δ*lldR*)/pBSPPc_Gm_-*vgb*	90.85 ± 0.75	91.9	1.43 ± 0.002

The initial dl-lactate concentration was 100 mM. The biocatalysis reactions were conducted at 30 °C in phosphate buffer (pH 7.4) for 6 h with 10.5 g DCW/L of biocatalysts prepared from glycerol. Results are mean ± SD of three parallel replicates

^a^The yields of pyruvate were calculated based on the actual initial concentrations of dl-lactate measured by HPLC

**Table 3:** Comparison of 2-OBA productions by whole cells of *P. putida* KT2440, *P. putida* KT2440/pBSPPc_Gm_-*vgb*, *P. putida* KT2440 (Δ*lldR*), and *P. putida* KT2440 (Δ*lldR*)/pBSPPc_Gm_-*vgb*

Strain	2-OBA (mM)	Yield (%)^a^	Productivity (mmol/g DCW h)
KT2440	0	0	0
KT2440/pBSPPc_Gm_-*vgb*	0	0	0
KT2440 (Δ*lldR*)	75.52 ± 1.59	74.7	1.20 ± 0.03
KT2440 (Δ*lldR*)/pBSPPc_Gm_-*vgb*	99.31 ± 1.45	99.8	1.58 ± 0.02

The initial dl-2-HBA concentration was 100 mM. The biocatalysis reactions were conducted at 30 °C in phosphate buffer (pH 7.4) for 6 h with 10.5 g DCW/L of biocatalysts prepared from glycerol. Results are mean ± SD of three parallel replicates

^a^The yields of 2-OBA were calculated based on the actual initial concentrations of dl-2-HBA measured by HPLC

Many researches have focused on the conversions of the inexpensive glycerol into high-value products, such as fine chemicals [6] and biodiesels [16], via the microbial fermentation. Furthermore, glycerol can also be used as a carbon source and energy source to support the growth of many industrial microorganisms [8]. The development of biotechnology of glycerol utilization processes will allow the biofuel industry to be more competitive.

**Fig. 3:**
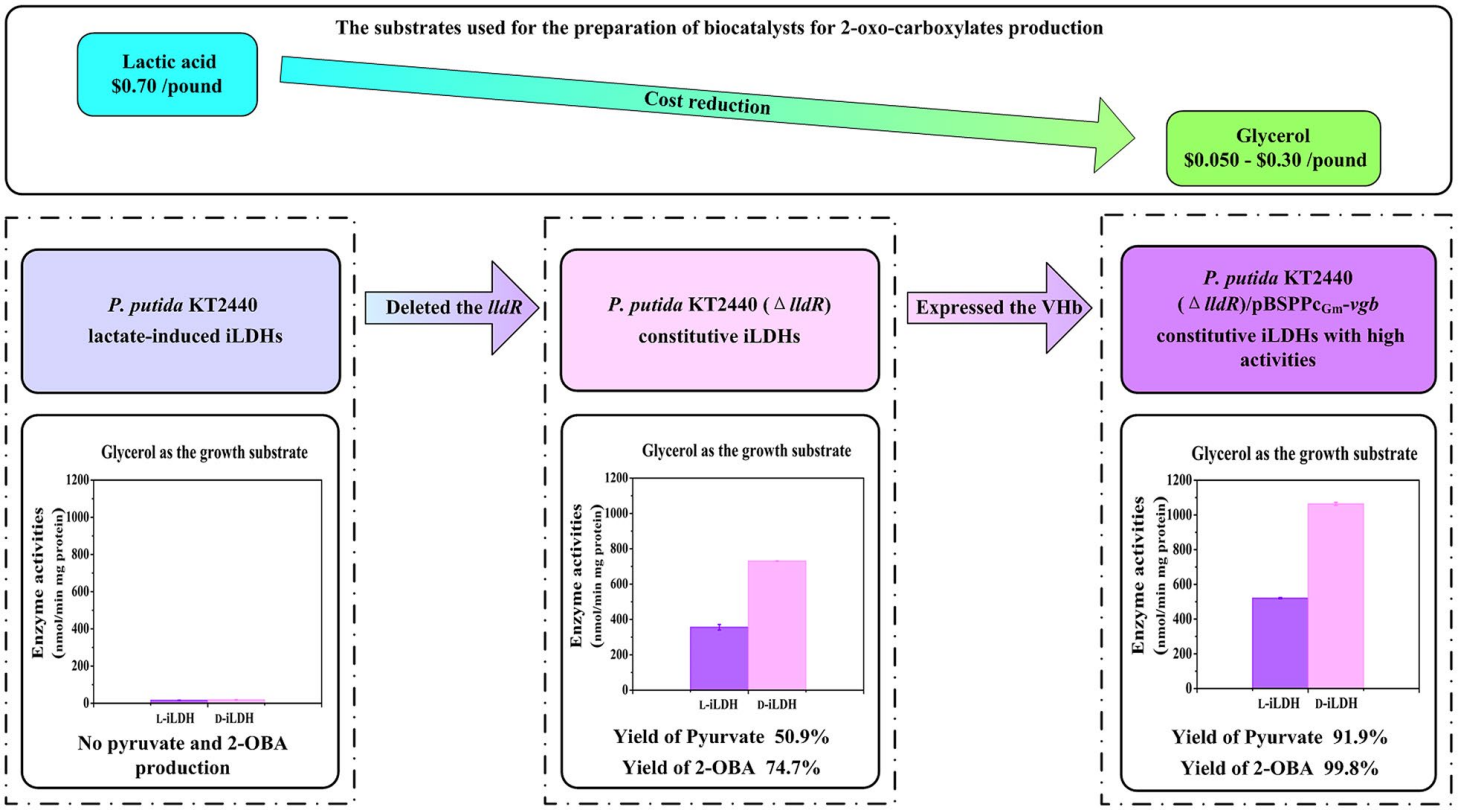
The optimization process of the biocatalysts prepared with glycerol

Compared with lactate, glycerol is a more cost-effective green substrate suitable for preparation of the biocatalyst containing the iLDHs. In 2006 and 2007, the spot price of the heat-stable lactic acid, as posted in the Chemical Marketing Reporter, was about $0.70 per pound [48]. In contrast, the prices of refined glycerol and crude glycerol were approximately $0.30 and $0.050 per pound, respectively, obviously cheaper than the lactic acid [49]. Furthermore, as a byproduct of biofuels, large amounts of glycerol have become a waste stream. Making good use of this waste stream would not only lower the production costs, but also contribute to the sustainable development of the biofuels industry. After reconstructing the lactate utilization regulatory network by deleting the LldR regulator in *P. putida* KT2440, the Δ*lldR* mutant exhibited the outstanding constitutive iLDHs activities when cultured with glycerol as the carbon source (Fig. 3). On the other hand, a constitutive vector with high expression strengths and broad host ranges was used for the introduction of VHb, which can enhance the lactate oxidation activities and the yields of the 2-oxo-carboxylates significantly (Fig. 3). This beneficial effect of VHb heterologous expression may be the result of binding more oxygen and delivering to the respiratory chain [43]. It is also feasible to apply this VHb introduction technology in other aerobic biotransformations to increase production efficiency.

Pyruvate and 2-OBA are two important platform compounds which have been widely applied in the chemical, drug, and food industries [47, 50]. Pyruvate has been used as a weight-control dietary supplement, a supplemental nutrient, and an antioxidant which can protect the brain and other tissues from the oxidative stress [50–53]. 2-OBA is an important intermediate widely used in biosynthesis of l-isoleucine, d-2-hydroxybutyrate and 1-propanol [54–56]. Furthermore, 2-OBA could be bioconverted into a non-natural amino acid l-homoalanine, which is a key chiral precursor for production of levetiracetam, brivaracetam, and ethambutol [57, 58]. Owing to the mild reaction conditions, high substrate conversion efficiency and simple compositions of the reaction mixture which is contributed to the convenience of recovery, whole cell catalysis becomes the preferred method for pyruvate and 2-OBA production [47, 50]. For example, *Pseudomonas stutzeri* SDM, which contains inducible iLDHs, has been reported to have good ability to produce pyruvate and 2-OBA from lactate and 2-HBA as the substrates, respectively [37, 47]. Whole cells of *P. stutzeri* SDM with iLDHs must be prepared with lactate as the carbon source. Although the concentrations of pyruvate and 2-OBA reported here were lower than these previous reports, this work disclosed a novel biocatalyst which could be prepared with glycerol as a more cost-effective substrate.

## Conclusions

Taking the 2-oxo-carboxylates production as an example, we developed a process of using biofuel’s byproduct glycerol for biocatalyst preparation. After deleting the LldR and heterologously expressing VHb in *P. putida* KT2440, the recombinant *P. putida* KT2440 (Δ*lldR*)/pBSPPc_Gm_-*vgb* with high iLDHs activities was cost-effectively prepared from glycerol. Using the whole cells as biocatalyst, 90.9 mM pyruvate and 99.3 mM 2-OBA were produced in 6 h from 100 mM dl-lactate and dl-2-HBA, respectively. The process demonstrated an option for effective utilization of the low-cost and renewable substrates through recombining the metabolic networks, based on the regulation mechanism, with the goal of producing the high-value chemicals.

## Methods

### Chemicals and biochemicals

Racemic lactate, l-lactate, octanoate, phenylmethanesulfonyl fluoride (PMSF), and DCIP were all purchased from Sigma. d-Lactate and racemic 2-HBA were obtained from Fluka and TCI, respectively. Restriction enzymes and T4 DNA ligase were purchased from Thermo Fisher Scientific Inc. FastPfu DNA polymerase was purchased from TransGen Biotech. All other chemicals were of reagent grade.

### Bacterial strains and culture conditions

Bacterial strains, plasmids, and oligonucleotide primers used in this study are listed in Table 4. For DNA manipulations and for pre-cultures, *E. coli* and *P. putida* strains were grown in Luria–Bertani (LB) medium [59, 60] at 37 and 30 °C, respectively. Antibiotics at the following concentrations: kanamycin at 50 μg/mL, ampicillin at 100 μg/mL, and gentamicin at 30 μg/mL were added when necessary. For determination of iLDHs activities in crude cell extracts and preparation of whole-cell catalysts, *P. putida* KT2440 and its derivatives were inoculated into 500-mL baffled shake flasks each containing 100 mL MSM supplemented with 5 g/L dl-lactate or glycerol as the carbon source at 30 °C and 120 rpm for 12 h, respectively. And 1 mM octanoate was added to the glycerol MSM as the co-feeder to shorten lag phase [23].

**Table 4:** Strains, plasmids, and oligonucleotide primers used in this study

Name	Relevant characteristic	Reference
Strains		
*P. putida* KT2440	Wild-type; capable of dl-lactate utilizing	ATCC
*P. putida* KT2440 (Δ*lldR*)	*lldR* deletion strain of *P. putida* KT2440	This study
*P. putida* KT2440/pBSPPc_Gm_-*vgb*	*P. putida* KT2440 harboring pBSPPc_Gm_-*vgb*; Gm^r^	This study
*P. putida* KT2440 (Δ*lldR*)/pBSPPc_Gm_-*vgb*	*P. putida* KT2440 (Δ*lldR*) harboring pBSPPc_Gm_-*vgb*; Gm^r^	This study
*E. coli* DH5α	λ^−^ ϕ80*lacZ*∆M15 ∆ (*lacZYA*-*argF*)* U169 recA1 endA1 hsdR17* (r_K_^−^, m_K_^+^) *supE44 thi*-*1* *gyrA relA1*; used for gene clone	Invitrogen
*E. coli* BL21 (pET28b-RgDAAO-VHb)	*E. coli* BL21 harboring pET28b-RgDAAO-VHb; Km^r^	Professor Sheng Yang^a^
Plasmids		
pK18*mobsacB*	Allelic exchange vector, *ori*ColE1 Mob^+^, *lacZα*, *sacB*; Km^r^	[36]
pKLR	A fragment from KT2440 genome containing whole length of *lldR* was inserted in pK18*mobsacB*; Km^r^	This study
pKSR	pKLR was completely digested by *pst*I, and then the large fragment was self-ligated; as a result, only partial length of *lldR* was inserted into pK18*mobsacB*; Km^r^	This study
pBSPPc_Gm_	A constitutive vector with high expression strengths; Gm^r^	[44]
pET28b-RgDAAO-VHb	pET28b containing *Rg*-*daao* gene and *vgb* gene,T7 promoter; Km^r^	Professor Sheng Yang^a^
pBSPPc_Gm_-*vgb*	pBSPPc_Gm_ containing gene *vgb*; Gm^r^	This study
Primers	Sequences (5′ → 3′) and properties	
*lldR*k.f	GAATTCATGGTTTTTGATCAGGTACGC (*EcoR*I)	This study
*lldR*k.r	AAGCTTTCAGCGCCCGCTGCGCCGCTCT (*Hind*III)	This study
*vgb*.f	CCCAAGCTTAGGAGACAGTAATGTTAGACCAGCAAACCATTA (*Hind*III)	This study
*vgb*.r	CGCGGATCCTTCAACCGCTTGAGCGTA (*BamH*I)	This study

*ATCC* American Type Culture Collection

^a^Form Institute of Plant Physiology and Ecology, Shanghai Institutes for Biological Sciences, Chinese Academy of Sciences

### Gene knockout procedure

Genomic DNA of *P. putida* KT2440 was extracted through the Wizard Genomic DNA Purification Kit (Promega, Madison, WI, USA). The *lldR* gene encoding the regulator LldR was amplified by PCR using *lldR*k.f and *lldR*k.r as the primers with the genomic DNA of *P. putida* KT2440 as the template, and then cloned into *Eco*RI and *Hin*dIII digested pK18*mobsacB* to form a new plasmid pKLR. Plasmid pKLR was completely digested by *Pst*I, and the large fragment was self-ligated using T4 DNA ligase to form pKSR that contained the deleted version of *lldR*. After being transferred into *P. putida* KT2440 by electroporation, the first crossover cells containing the integration of the plasmid pKSR into the chromosome of *P. putida* KT2440 were selected on LB plate supplemented with 50 μg/mL kanamycin. The second crossover cells were singled out by culture on LB plates containing 10 % (w/v) sucrose [22]. The resulting deletion mutant was designated as *P. putida* KT2440 (Δ*lldR*). All the constructed strains were validated by PCR and DNA sequencing.

### Cloning and expression of VHb in *P. putida* KT2440 and its derivatives

For the construction of VHb expression plasmid, VHb encoding gene (*vgb*) was amplified by PCR using a pair of primers, *vgb*.f and *vgb*.r, with plasmid pET28b-RgDAAO-VHb as a template. The PCR product was first ligated to the p*EASY*-Blunt vector, and the resulting plasmid was named p*EASY*-Blunt-*vgb* and sequenced. And then, after digested with *Hind*III and *BamH*I, the gel-purified *vgb* fragment was cloned into pBSPPc_Gm_, a constitutive vector with high expression strengths [44], at the corresponding sites, to result the plasmid pBSPPc_Gm_-*vgb*. Plasmid pBSPPc_Gm_-*vgb* was transferred into *P. putida* KT2440 and its derivatives by electroporation. The *P. putida* KT2440 and its derivatives, which harboring the recombinant vector pBSPPc_Gm_-*vgb* were selected on LB plate supplemented with 30 μg/mL gentamicin.

### Biocatalyst preparation

Whole cells of *P. putida* strains cultured in MSM containing 5 g/L glycerol and 1 mM octanoate were harvested by centrifugation at a speed of 6000 rpm for 10 min. After washing twice with phosphate buffer (pH 7.4), whole cells were suspended in phosphate buffer and ready for the following study. Optical densities of whole cells were assayed and converted to DCW according to the previous report [22].

### Enzymatic activity assays

Whole cells of the *P. putida* strains grown in MSM containing 5 g/L glycerol and 1 mM octanoate were disrupted by sonication (Sonics 500 W/20 kHz, USA) on ice bath. The disrupted cells were centrifuged for 20 min at 12,000 rpm, and the supernatants were used as crude cell extracts [37]. The activities of iLDHs were assayed by monitoring the change in absorbance at 600 nm corresponding to the reduction of DCIP at 30 °C with a UV/visible spectrophotometer (Ultrospec 2100 pro, Amersham Biosciences, USA) [61]. The reaction was carried out in 0.8 mL of 50 mM Tris–HCl, pH 7.5, containing 20 mM l- or d-lactate, 0.0625 mM DCIP, and 50 μL of crude cell extracts [22].

### Whole-cell biocatalysis for production of pyruvate and 2-OBA

The reactions were carried out at 30 °C and 120 rpm in phosphate buffer (pH 7.4) containing 10.5 g DCW/L of biocatalysts, 30 mM EDTA, and 100 mM racemic 2-hydroxy-carboxylates. The concentrations of pyruvate and 2-OBA in the reaction mixtures were quantitatively analyzed by high-performance liquid chromatography (HPLC) using an Aminex HPX-87H column (Bio-Rad) and the mobile phase (10 mM H_2_SO_4_) at 0.4 mL/min flow rate at 55 °C [37].

## Abbreviations

iLDHs: NAD-independent lactate dehydrogenases
2-OBA: 2-Oxobutyrate
HTH: Helix-turn-helix
WHTH: Winged HTH
G3P: sn-glycerol-3-P
VHb: *Vitreoscilla* hemoglobin
MSM: Minimal salt medium
DCW: Dry cell weight
EDTA: Ethylenediaminetetraacetic acid
2-HBA: 2-Hydroxybutyrate
PMSF: Phenylmethanesulfonyl fluoride
DCIP: 2,6-Dichloroindophenol
LB: Luria–Bertani
HPLC: High-performance liquid chromatography

## References

[1] Clomburg JM, Gonzalez R (2013). Anaerobic fermentation of glycerol: a platform for renewable fuels and chemicals. Trends Biotechnol.

[2] Yazdani SS, Gonzalez R (2007). Anaerobic fermentation of glycerol: a path to economic viability for the biofuels industry. Curr Opin Biotechnol.

[3] Johnson DT, Taconi KA (2007). The glycerin glut: options for the value-added conversion of crude glycerol resulting from biodiesel production. Environ Prog.

[4] Mattam AJ, Clomburg JM, Gonzalez R, Yazdani SS (2013). Fermentation of glycerol and production of valuable chemical and biofuel molecules. Biotechnol Lett.

[5] Posada JA, Rincón LE, Cardona CA (2012). Design and analysis of biorefineries based on raw glycerol: addressing the glycerol problem. Bioresour Technol.

[6] Katryniok B, Kimura H, Skrzyńska E, Girardon J-S, Fongarland P, Capron M (2011). Selective catalytic oxidation of glycerol: perspectives for high value chemicals. Green Chem.

[7] Khanna S, Goyal A, Moholkar VS (2012). Microbial conversion of glycerol: present status and future prospects. Crit Rev Biotechnol.

[8] da Silva GP, Mack M, Contiero J (2009). Glycerol: a promising and abundant carbon source for industrial microbiology. Biotechnol Adv.

[9] Nikel PI, Kim J, de Lorenzo V (2014). Metabolic and regulatory rearrangements underlying glycerol metabolism in *Pseudomonas putida* KT2440. Environ Microbiol.

[10] Metsoviti M, Paraskevaidi K, Koutinas A, Zeng AP, Papanikolaou S (2012). Production of 1,3-propanediol, 2,3-butanediol and ethanol by a newly isolated *Klebsiella oxytoca* strain growing on biodiesel-derived glycerol based media. Process Biochem.

[11] Yen HW, Li FT, Chang JS (2014). The effects of dissolved oxygen level on the distribution of 1,3-propanediol and 2,3-butanediol produced from glycerol by an isolated indigenous *Klebsiella* sp. Ana-WS5. Bioresour Technol.

[12] Huang Y, Li Z, Shimizu K, Ye Q (2012). Simultaneous production of 3-hydroxypropionic acid and 1,3-propanediol from glycerol by a recombinant strain of *Klebsiella pneumoniae*. Bioresour Technol.

[13] Almeida JR, Fávaro LC, Quirino BF (2012). Biodiesel biorefinery: opportunities and challenges for microbial production of fuels and chemicals from glycerol waste. Biotechnol Biofuels.

[14] Liang MH, Jiang JG (2013). Advancing oleaginous microorganisms to produce lipid via metabolic engineering technology. Prog Lipid Res.

[15] Munch G, Sestric R, Sparling R, Levin DB, Cicek N (2015). Lipid production in the under-characterized oleaginous yeasts, *Rhodosporidium babjevae* and *Rhodosporidium diobovatum*, from biodiesel-derived waste glycerol. Bioresour Technol.

[16] Yang L, Zhu Z, Wang WH, Lu XF (2013). Microbial recycling of glycerol to biodiesel. Bioresour Technol.

[17] Bornscheuer UT, Huisman GW, Kazlauskas RJ, Lutz S, Moore JC, Robins K (2012). Engineering the third wave of biocatalysis. Nature.

[18] Nikel PI, Martínez-García E, de Lorenzo V (2014). Biotechnological domestication of pseudomonads using synthetic biology. Nat Rev Microbiol.

[19] Gao C, Hu C, Zheng Z, Ma C, Jiang T, Dou P (2012). Lactate utilization is regulated by the FadR-type regulator LldR in *Pseudomonas aeruginosa*. J Bacteriol.

[20] Jiménez JI, Miñambres B, García JL, Díaz E (2002). Genomic analysis of the aromatic catabolic pathways from *Pseudomonas putida* KT2440. Environ Microbiol.

[21] Park SJ, Choi JS, Kim BC, Jho SW, Ryu JW, Park D (2009). PutidaNET: interactome database service and network analysis of *Pseudomonas putida* KT2440. BMC Genom.

[22] Wang Y, Lv M, Zhang Y, Xiao X, Jiang T, Zhang W (2014). Reconstruction of lactate utilization system in *Pseudomonas putida* KT2440: a novel biocatalyst for l-2-hydroxy-carboxylate production. Sci Rep.

[23] Escapa IF, del Cerro C, García JL, Prieto MA (2013). The role of GlpR repressor in *Pseudomonas putida* KT2440 growth and PHA production from glycerol. Environ Microbiol.

[24] Nikel PI, Romero-Campero FJ, Zeidman JA, Goñi-Moreno Á, de Lorenzo V. The glycerol-dependent metabolic persistence of *Pseudomonas putida* KT2440 reflects the regulatory logic of the GlpR repressor. mBio. 2015;6:e00340–15. 10.1128/mBio.00340-15 PMC4453509 25827416

[25] Zeng G, Ye S, Larson TJ (1996). Repressor for the sn-glycerol 3-phosphate regulon of *Escherichia coli* K-12: primary structure and identification of the DNA-binding domain. J Bacteriol.

[26] Larson TJ, Ye SZ, Weissenborn DL, Hoffmann HJ, Schweizer H (1987). Purification and characterization of the repressor for the sn-glycerol 3-phosphate regulon of *Escherichia coli* K12. J Biol Chem.

[27] Škerlová J, Fábry M, Hubálek M, Otwinowski Z, Rezáčová P (2014). Structure of the effector-binding domain of deoxyribonucleoside regulator DeoR from *Bacillus subtilis*. FEBS J.

[28] Gao C, Jiang T, Dou P, Ma C, Li L, Kong J (2012). NAD-independent l-lactate dehydrogenase is required for l-lactate utilization in *Pseudomonas stutzeri* SDM. PLoS One.

[29] Jiang T, Gao C, Su F, Zhang W, Hu C, Dou P (2012). Genome sequence of *Pseudomonas stutzeri* SDM-LAC, a typical strain for studying the molecular mechanism of lactate utilization. J Bacteriol.

[30] Kemp MB (1972). d- and l-Lactate dehydrogenases of *Pseudomonas aeruginosa*. Biochem J.

[31] Ma C, Gao C, Qiu J, Hao J, Liu W, Wang A (2007). Membrane-bound l- and d-lactate dehydrogenase activities of a newly isolated *Pseudomonas stutzeri* strain. Appl Microbiol Biotechnol.

[32] Gao YG, Suzuki H, Itou H, Zhou Y, Tanaka Y, Wachi M (2008). Structural and functional characterization of the LldR from *Corynebacterium glutamicum*: a transcriptional repressor involved in l-lactate and sugar utilization. Nucleic Acids Res.

[33] Huffman JL, Brennan RG (2002). Prokaryotic transcription regulators: more than just the helix-turn-helix motif. Curr Opin Struct Biol.

[34] Jain D (2015). Allosteric control of transcription in GntR family of transcription regulators: a structural overview. IUBMB Life.

[35] Gao C, Xu X, Hu C, Zhang W, Zhang Y, Ma C (2010). Pyruvate producing biocatalyst with constitutive NAD-independent lactate dehydrogenases. Process Biochem.

[36] Schäfer A, Tauch A, Jäger W, Kalinowski J, Thierbach G, Pühler A (1994). Small mobilizable multi-purpose cloning vectors derived from the *Escherichia coli* plasmids pK18 and pK19: selection of defined deletions in the chromosome of *Corynebacterium glutamicum*. Gene.

[37] Gao C, Qiu J, Ma C, Xu P (2012). Efficient production of pyruvate from dl-lactate by the lactate-utilizing strain *Pseudomonas stutzeri* SDM. PLoS One.

[38] Nelson KE, Weinel C, Paulsen IT, Dodson RJ, Hilbert H, Martins dos Santos VA (2002). Complete genome sequence and comparative analysis of the metabolically versatile *Pseudomonas putida* KT2440. Environ Microbiol.

[39] Wakabayashi S, Matsubara H, Webster D (1986). Primary sequence of a dimeric bacterial haemoglobin from *Vitreoscil*la. Nature.

[40] Stark BC, Dikshit KL, Pagilla KR (2012). The biochemistry of *Vitreoscilla* hemoglobin. Comput Struct Biotechnol J.

[41] Khosla C, Bailey J (1988). Heterologous expression of a bacterial hemoglobin improves the growth properties of recombinant *Escherichia coli*. Nature.

[42] Dikshit KL, Webster D (1988). Cloning, characterization and expression of the bacterial globin gene from *Vitreoscilla* in *Escherichia coli*. Gene.

[43] Stark BC, Pagilla KR, Dikshit KL (2015). Recent applications of *Vitreoscilla* hemoglobin technology in bioproduct synthesis and bioremediation. Appl Microbiol Biotechnol.

[44] Xu Y, Tao F, Ma C, Xu P (2013). New constitutive vectors: useful genetic engineering tools for biocatalysis. Appl Environ Microbiol.

[45] Jiang T, Gao C, Dou P, Ma C, Kong J, Xu P (2012). Rationally re-designed mutation of NAD-independent l-lactate dehydrogenase: high optical resolution of racemic mandelic acid by the engineered *Escherichia coli*. Microb Cell Fact.

[46] Ma CQ, Xu P, Dou YM, Qu YB (2003). Highly efficient conversion of lactate to pyruvate using whole cells of *Acinetobacter* sp.. Biotechnol Prog.

[47] Gao C, Zhang W, Lv C, Li L, Ma C, Hu C (2010). Efficient production of 2-oxobutyrate from 2-hydroxybutyrate by using whole cells of *Pseudomonas stutzeri* strain SDM. Appl Environ Microbiol.

[48] Datta R, Henry M (2006). Lactic acid: recent advances in products, processes and technologies—a review. J Chem Technol Biotechnol.

[49] Yang F, Hanna MA, Sun R (2012). Value-added uses for crude glycerol–a byproduct of biodiesel production. Biotechnol Biofuels.

[50] Xu P, Qiu J, Gao C, Ma C (2008). Biotechnological routes to pyruvate production. J Biosci Bioeng.

[51] Koh-Banerjee PK, Ferreira MP, Greenwood M, Bowden RG, Cowan PN, Almada AL (2005). Effects of calcium pyruvate supplementation during training on body composition, exercise capacity, and metabolic responses to exercise. Nutrition.

[52] McCarty MF (2000). Toward a wholly nutritional therapy for type 2 diabetes. Med Hypotheses.

[53] Wang X, Perez E, Liu R, Yan LJ, Mallet RT, Yang SH (2007). Pyruvate protects mitochondria from oxidative stress in human neuroblastoma SK-N-SH cells. Brain Res.

[54] Eggeling L, Morbach S, Sahm H (1997). The fruits of molecular physiology: engineering the l-isoleucine biosynthesis pathway in *Corynebacterium glutamicum*. J Biotechnol.

[55] Krix G, Bommarius AS, Drauz K, Kottenhahn M, Schwarm M, Kula MR (1997). Enzymatic reduction of α-keto acids leading to l-amino acids, d- or l-hydroxy acids. J Biotechnol.

[56] Atsumi S, Hanai T, Liao JC (2008). Non-fermentative pathways for synthesis of branched-chain higher alcohols as biofuels. Nature.

[57] Zhang K, Li H, Cho KM, Liao JC (2010). Expanding metabolism for total biosynthesis of the nonnatural amino acid l-homoalanine. Proc Natl Acad Sci USA.

[58] Zhang W, Gao C, Che B, Ma C, Zheng Z, Qin T (2012). Efficient bioconversion of l-threonine to 2-oxobutyrate using whole cells of *Pseudomonas stutzeri* SDM. Bioresour Technol.

[59] Miller JH (1992). A short course in bacterial genetics: A laboratory manual and handbook for *Escherichia coli* and related bacteria.

[60] Sambrook J, Russell DW (2001). Molecular cloning: a laboratory manual.

[61] Armstrong JM (1964). The molar extinction coefficient of 2,6-dichlorophenol Indophenol. Biochim Biophys Acta.

